# New technologies as tools to prevent suicide in adolescence: A literature overview

**DOI:** 10.1192/j.eurpsy.2021.935

**Published:** 2021-08-13

**Authors:** G. Sarli, L. Polidori, A. Forte, M. Pompili

**Affiliations:** 1 Psychiatry Residency Training Program, Faculty Of Medicine And Psychology, Sapienza University of Rome, Roma, Italy; 2 Department Of Neurosciences, Mental Health And Sensory Organs, Suicide Prevention Center, Sant’andrea Hospital, Sapienza University Of Rome, Rome, Italy, Sapienza university of Rome, ROMA, Italy; 3 Department Of Neurosciences, Mental Health And Sensory Organs, Suicide Prevention Center, S. Andrea Hospital, Sapienza University, Rome, Italy

**Keywords:** Suicide, adolescence, Technology, e-mental health

## Abstract

**Introduction:**

Suicide in adolescents represents a major public health concern. To date, a growing number of suicide preventive strategies based on the use of new technologies are emerging.

**Objectives:**

The purpose of the present paper is to provide an overview of the present literature on the use of new technologies in adolescent suicide prevention.

**Methods:**

A systematic electronic search was run using the following keywords: Technology OR Technologies OR APP OR Application OR mobile application) AND (Adolescent OR youth OR puberty) AND (Suicid* OR Self-harm OR self-destruction).

**Results:**

We found 12 studies on the use of telemedicine, 7 on mobile applications, and 3 on language detection. Heterogeneity regarding the study design was found: 3 Randomized Controlled Trial (RCT), 13 are Open-label single group trials, 2 Randomized studies, and 1 Cross-sectional study. Telemedicine was the most adopted tool, especially web-based approaches. Mobile applications mostly focused on screening of depressive symptoms and suicidal ideation, and for clinical monitoring through the use of text messages.

**Conclusions:**

Despite telepsychiatry and mobile applications can provide a fast and safe tool, only a few studies demonstrated efficacy in preventing suicide among adolescents through the use of these interventions. Some studies suggested sophisticated algorithms able to recognize people at risk for suicide from language detection on social media posts. To date, only a few data support the use of such interventions in clinical practice and preventive strategies. Further studies are needed to test their efficacy in suicide prevention among adolescents and young adults.

